# Postprandial Glycemic and Insulinemic Response by a Brewer’s Spent Grain Extract-Based Food Supplement in Subjects with Slightly Impaired Glucose Tolerance: A Monocentric, Randomized, Cross-Over, Double-Blind, Placebo-Controlled Clinical Trial

**DOI:** 10.3390/nu14193916

**Published:** 2022-09-21

**Authors:** Hammad Ullah, Cristina Esposito, Roberto Piccinocchi, Lorenza Francesca De Lellis, Cristina Santarcangelo, Alessandro Di Minno, Alessandra Baldi, Daniele Giuseppe Buccato, Ayesha Khan, Gaetano Piccinocchi, Roberto Sacchi, Maria Daglia

**Affiliations:** 1 Department of Pharmacy, University of Naples Federico II, Via Domenico Montesano 49, 80131 Naples, Italy; 2 Level 1 Medical Director Anaesthesia and Resuscitation A. U. O. Luigi Vanvitelli, Via Santa Maria di Costantinopoli, 80138 Naples, Italy; 3 CEINGE-Biotecnologie Avanzate, Via Gaetano Salvatore 486, 80145 Naples, Italy; 4 Department of Medicine, Combined Military Hospital Nowshera, Nowshera 24110, Pakistan; 5 Comegen S.c.S., Societ‘a Cooperativa Sociale di Medici di Medicina Generale, Viale Maria Bakunin 41, 80125 Naples, Italy; 6 Applied Statistic Unit, Department of Earth and Environmental Sciences, University of Pavia, Viale Taramelli 24, 27100 Pavia, Italy; 7 International Research Center for Food Nutrition and Safety, Jiangsu University, Zhenjiang 212013, China

**Keywords:** brewer’s spent grains, clinical trial, dietary fiber, food supplement, insulinemia, postprandial glycemia

## Abstract

Dietary fiber exerts beneficial effects on human health reducing the risk factors of metabolic related diseases such as hyperglycemia, insulin resistance, and hypercholesterolemia. The aim of this study is to demonstrate the efficacy of a food supplement based on brewer’s spent grain (BSG) extract in the reduction of postprandial glycemia and insulinemia in normoglycemic subjects. BSG was chemically characterized, revealing the presence of resistant starch (14.64 g/100 g), arabinoxylans (7.50 g/100 g), β-glucans (1.92 g/100 g) and other soluble fibers (6.43 g/100 g), and bioaccessible ferulic acid (91.3 mg/100 g). For the clinical study, 40 normoglycemic subjects were randomized into two groups, 1 and 2 (*n* = 20), for a cross-over clinical design and received either BSG extract-based food supplement or placebo. Postprandial blood glucose values were significantly lower than corresponding values in the placebo group after 90 and 120 min, while at the baseline and in the first 60 min, the two glycemic curves overlapped substantially. This improved clinical outcome was corroborated by significant reductions in postprandial insulinemia. None of the subjects reported adverse effects. This study showed that the tested BSG extract-based food supplement improves glucose metabolism and insulinemic response in normoglycemic subjects with at most a mild insulin resistance.

## 1. Introduction

A large body of evidence suggests that dietary fiber, especially soluble fiber, exerts beneficial effects on human health, as it may reduce the risk of cardiovascular diseases, metabolic related issues, including diabetes and obesity, gastrointestinal ailments, and cancer along with the improvement of mental health and other cognitive functions [1,2,3,4]. The effects of dietary fiber can be exerted both directly, through the reduction of lipid and glucose absorption, which in turn decreases blood lipids and maintains blood glucose levels, and indirectly through its prebiotic effect, which leads to the growth of eubiotic bacteria (i.e., Blautia, Roseburia, and Turicibacter) producing short chain fatty acids (SCFAs, i.e., acetic acid, propionic acid, and butyric acid), which improve intestinal permeability, insulin sensitivity, and relieve inflammation and glucose intolerance [5].

Among the various dietary fiber categories, arabinoxylans (AXs) are non-starch polysaccharides composed of a central linear carbonaceous skeleton consisting of β-1,4-linked xylose monomers (D-xylopyranose) and, as substituents, arabinose (L-arabinofuranose) side chains, which may have a ferulic acid on the fifth carbon. The covalent bond between ferulic acid residues is primarily responsible for the formation of gels, which in turn are responsible for simple sugar and lipid absorption reduction [6]. The main sources of AXs are wheat, barley, rice, rye, oats, and sorghum [7]. AXs, based on their structural and conformational properties, are classified into water extractable and non-water extractable AXs. The ratio between the two types of compounds varies depending on the species, cultivar, type of caryopsis tissue (bran, endosperm), external environment, and processing or extraction process [8]. AXs cannot be degraded by mammalian enzymes present in the digestive tract but are degraded by the gut microbiota into SCFAs and other products [9]. In a recent study by Lynch et al., soluble arabinoxylan extracted from brewer’s spent grains (BSG) showed prebiotic effects, resulting from 2-fold and 3.5-fold increases in *Lactobacillus* and bifidogenic levels, respectively [10]. AXs also possess antioxidant capacity, with higher antioxidant activity when ferulic acid is bound [11,12].

In recently published studies, water-soluble AXs from wheat have been found to be effective in modulating the metabolism of glucose. The mechanisms of action that contribute to reducing glucose absorption and attenuating the postprandial glycemic response involve delaying gastric emptying time, slowing intestinal transit, reducing the glucose diffusion rate in the intestinal lumen, lowering the availability and inhibiting the activity of digestive enzymes in the intestinal lumen, and the bifidogenic effect [13,14,15,16]. In addition, the gelling properties of AXs delay the degradation and, consequently, the absorption of proteins by attenuating the insulinemic response due to insulinogenic amino acids [17]. In view of such properties, wheat endosperm AXs have been granted a European health claim for reducing postprandial blood glucose [18].

The growing interest in food fiber and especially AXs has led research towards the development of new ingredients to be used in the production of functional foods and food supplements using by-products of the agri-food industry to develop products that are economically and environmentally sustainable. In keeping with this, as BSG is a by-product of the brewing industry rich in insoluble fiber (i.e., cellulose, hemicellulose, and lignin) and soluble dietary fibers, especially AXs, it is an ideal candidate to be exploited as a raw material from which to obtain extracts with a high fiber content [19]. Nowadays, 70% of BSG are used as animal feed, 10% are used for biogas production, and the remaining 20% are disposed of in landfills [20]. Considering that about 3.4 and 4.5 million tons of BSG are generated in Europe and the USA, respectively, and a medium-sized brewery produces about 7–8 tons of BSG/week, the enhancement of these by-products to obtain food ingredients rich in fiber for the food industry with high added value is another possible way to utilize BSG [21]. 

Thus, the aim of this study is the evaluation of the properties of reducing post-prandial glycemia and improving insulinemia response of a food supplement containing a BSG extract chemically characterized in terms of total fibers, AXs, β-glucans, resistant starch, total polyphenols, and ferulic acid in healthy subjects through a randomized, cross-over, double-blind, placebo-controlled clinical trial.

## 2. Materials and Methods

### 2.1. BSG Extract-Based Food Supplement, and Placebo Used in the Clinical Study

BSG extract-based food supplement and placebo were produced by HEALLO s.r.l. (Milan, Italy), within European specifications for contaminants and microbiologic limits. The food supplement has been notified to the Italian Health Ministry, with the brand name “JAX Plus^®^” (notification number: 141039). The BSG extract-based food supplement, in the form of soluble granules in single-dose stick packs (5 g), contains 5.0 g/stick pack of BSG extract, which consist of 4.25 g of BSG extract and 0.75 g (corresponding to 15%) of inulin used as carrier agent. Placebo in the same form consisted of microcrystalline cellulose and the same amounts of inulin (15%). The bakery product (breadsticks) consumed by the subjects recruited in the present clinical study was previously portioned and packaged (net weigh 65 g) in such a quantity as to provide 50 g of available carbohydrates and was characterized in terms of nutrients and caloric value (Table 1).

### 2.2. Chemical Characterization of BSG Extract

#### 2.2.1. Total Dietary Fiber Determination

Total dietary fiber content (TDF) was determined using the Total Dietary Fiber Assay Kit (Neogen, Lansing, MI, USA) according to the manufacturer’s protocol [22], which represents a simplified version of the official AOAC 985.29 method for the determination of total fiber. TDF was determined on quintuplicate samples. Five aliquots of BSG extract (1.000 ± 0.005 g) were incubated at about 100 °C with 50 µL heat-stable α-amylase solution to allow starch gelatinization, hydrolysis, and depolymerization. The samples were then incubated at 60 °C with 100 µL protease solution (to solubilize and depolymerize the proteins) and 200 µL amyloglucosidase solution (to hydrolyze the starch fragments into glucose). The samples were filtered to separate the insoluble fiber. The supernatants were then treated with approximately four volumes of ethanol to allow precipitation of the soluble fibers and remove the depolymerized proteins and glucose (from starch). The samples were then filtered and the residues, corresponding to the soluble fiber, were washed with 78% ethanol, 95% ethanol, and acetone, and dried overnight in a microwave oven at 103 °C and then weighed. The first residue was analyzed for proteins, determined using the Bradford method [23], and the second one was incubated at 525 °C to determine the ash content. TDF was calculated as the sum of the weight of insoluble fiber and soluble fiber dry residue minus protein and ash weights.

The other three residues were stored at room temperature pending analysis to determine the content of AXs, β-glucans, and resistant starch.

#### 2.2.2. Glucose, Arabinose, Xylose, Total Β-Glucans, Total Arabinoxylans, and Resistant Starch Determination

According to the manufacturer’s protocol, the BSG extract and the three soluble fiber dry residues previously described, obtained in the total dietary fiber assay, were prepared for carrying out the determination of (1) free glucose, (2) glucose deriving from rapidly digestible starch and slowly digestible starch, (3) glucose from resistant starch, (4) arabinose, (5) xylose, (6) AXs, and (7) β-glucans using the commercially available kits for the measurements of xylose, arabinose, glucose, and mixed linkage β-glucan (Neogen, Lansing, MI, USA). 

As far as the BSG extract is concerned, glucose, arabinose, and xylose, present in the extract before hydrolysis, were quantified by spectrophotometric analysis [24,25], using three calibration curves prepared with standard compounds at known concentrations. The results of these analyses provided the content of free simple sugars originally present in BSG extract. Then, BSG extract was submitted to hydrolysis with α-amylase and α-glucosidase at 60 °C for 30 min and for 4 h to obtain the content of glucose derived from rapidly digestible starch and slowly digestible starch and from resistant starch, respectively. Subsequently, glucose, and arabinose and xylose present in the extract after the hydrolysis of β-glucans and AXs, respectively, were quantified by spectrophotometric analysis. BSG extract β-glucan and AX concentrations were calculated on the basis of glucose, and arabinose and xylose concentrations, minus free glucose, and free arabinose and xylose, originally present in the extract before the enzymatic hydrolysis. 

With regards to the soluble fiber dry residues obtained from the total fiber assay, glucose, and arabinose and xylose, present in the dry residue after the hydrolysis of β-glucans, AXs, and resistant starch, respectively, were quantified by spectrophotometric analysis, and β-glucan, AX, and resistant starch concentrations were calculated on the basis of glucose, and arabinose and xylose concentrations.

#### 2.2.3. Water and Alkali Extractable Arabinoxylans

The isolation of the water extractable arabinoxylans (WEAX) and alkali extractable arabinoxylans (AEAX) in BSG extract was performed using a method from Buksa et al. [26]. After isolation, the quantification of WEAX and AEAX was performed according to the method reported in Section 2.2.2.

#### 2.2.4. Total Polyphenol Content and Ferulic Acid Determinations

##### Simulated In Vitro Oral-Gastric-Duodenal Digestion Process of BSG Extract

The simulated in vitro oral-gastric-duodenal digestion of BSG extract was performed following the protocol by Minekus et al. with some modifications [27]. The used reagents are reported below: potassium chloride (KCl), dihydrogen potassium phosphate (KH_2_PO_4_), sodium carbonate (NaHCO_3_), magnesium chloride (MgCl_2_), ammonium carbonate (NH_4_)CO_3_, calcium chloride (CaCl_2_), sodium chloride (NaCl), hydrochloric acid (HCl), and sodium hydroxide (NaOH). All were provided by Carlo Erba (Milan, Italy). Pancreatin from porcine pancreas (extract of pig bile), α-amylase from *Bacillus licheniformis*, pepsin from porcine gastric mucosa and porcine bile extract, formic acid solution (1 M), water, methanol, and acetonitrile LC–MS grade were sourced from Sigma-Aldrich, Merck KGaA (Milan, Italy). 

In brief, 5 g of the BSG extract were dissolved in 3.5 mL of previously prepared simulated salivary fluid (SSF). Then, 0.5 mL (1500 U/mL) of fresh α-amylase solution were added to both samples. In the end, water was added to reach a final volume of 10 mL and the sample was incubated for 2 min at 37 °C. The bolus obtained in the previous phase was mixed with 7.5 mL of simulated gastric fluid (SGF) and 1.6 mL (25,000 U/mL) of fresh pepsin; the pH was then adjusted to 2.00 ± 0.02 using 1 M HCl. The sample was brought up to 20 mL volume and the mixture was incubated at 37 °C for 2 h in a shaking water bath. Subsequently, gastric chyme was incubated with 5 mL of fresh pancreatin (800 U/mL) and 2.5 mL of fresh bile mixture (160 mM) to reach a final volume of 32.5 mL. The sample was finally made up to a 40 mL final volume; the pH was adjusted to 7.00 ± 0.02 using 1 M NaOH and incubated at 37 °C for 2 h. At the end of the digestion process, the oral-gastric-duodenal digested sample was freeze dried and maintained at 4 °C pending total polyphenol content determination and RP-HUPLC-MS analysis.

##### Total Polyphenol Content

Total polyphenol content (TPC) of the BSG extract and the freeze-dried digested samples (1 mg) was determined using a Folin–Ciocalteu method (colorimetric assay), with slight modifications [28]. A 10 µL aliquot of one of the sample solutions, namely the BSG extract, digested BSG extract (50 mg/mL), and gallic acid standard solutions (at concentrations ranging from 200 to 1000 µg/mL), was added to 50 µL of Folin–Ciocalteu reagent. The solutions were cyclomixed for 4 min, followed by the addition of 15% Na_2_CO_3_ (200 µL). Distilled water was added to the mixture to make the final volume up to 1 mL, and then allowed to incubate at room temperature for 2 h. The absorbance of the mixtures was read at the wavelength of 750 nm and the results were expressed as mg equivalent to gallic acid/g (GAE/g) of the extract on a dry weight basis.

##### UHPLC-MS Analysis and Quantification of Ferulic Acid after Digestion

BSG extract freeze-dried digested samples (1 mg) was solubilized in 1 mL of water and treated with 3 mL of ice-cold acetonitrile. The sample was centrifuged for 15 min at 16,000 rpm (Hermle^®^, Hamburg, Germany), and the solvent was evaporated with nitrogen; the supernatant was solubilized in 50:50 methanol/water 0.1% formic acid and analyzed. 

HUPLC-MS/MS analyses for the identification and quantification of ferulic acid in the digested sample were performed on a Dionex Ultimate 3000 UHPLC system (Thermo Fisher Scientific, Milan, Italy) consisting of two pumps, an autosampler, and a column oven. The system was coupled to a Linear Ion Trap Mass Spectrometer LTQ XL (Thermo Fisher Scientific, Milan, Italy) equipped with an electrospray source (ESI). 

The separation was performed on a LUNA^®^ OMEGA POLAR C18 column (L × ID) 150 mm × 2.1 mm, 3 μm (Phenomenex, Milan, Italy). The mobile phases used were A) 0.1% HCOOH in H_2_O and B) ACN, with the following gradient: 0–5 min, 2% B; 5–15 min, 2–18% B; 15–30 min 18–95% B and hold for 5 min; returning to initial conditions in 0.2 min. The flow rate was set to 0.3 mL/min. Column oven was set to 45 °C, and 5 μL of extract were injected. 

The ESI source was operated in the negative mode. MS/MS analyses were conducted in full mode, using the following ion with m/z 195.2 corresponding to ferulic acid, collision energy: 35.0 V, and scan range m/z 50–200. To optimize the MS operating conditions, a preliminary experiment was performed: 10 μg/mL ferulic acid (H_2_O/MeOH: 50/50 with 0.1% formic acid) solutions were directly infused in the ESI interface at a flow rate of 25 μL/min into the mass spectrometer. Optimized conditions were as follows: sheath gas 60, capillary temperature 220 °C, auxiliary gas 25, spray voltage 4.5 kV, and capillary voltage −26.13 V for negative ionization mode. Ferulic acid (Sigma Aldrich, Milan, Italy) was selected as the external standard for the quantitation. Stock solution (1 mg/mL) was prepared in H_2_O/MeOH: 50:50 with 0.1% formic acid, and the calibration curve (y = 3669.9x − 50.911) was obtained in the concentration range of 0.62–1 µg/mL (R^2^ = 0.9973).

### 2.3. Clinical Trial Design

A monocentric, randomized, placebo-controlled, double-blind, cross-over clinical trial was performed by COMEGEN—Società Cooperativa Sociale (Naples, Italy) to evaluate the effects of the target food supplement based on BSG extract on the reduction of postprandial glycemic response in normoglycemic subjects. The study was double blind, both for the investigating physician and for the enrolled subjects. For this purpose, both the food supplement containing dietary fiber obtained from BSG extract and the placebo were made to be unrecognizable in shape, weight, color, and, as far as possible, in taste. 

The participants received oral and written information regarding the study before they gave their written consent. Protocol, letter of intent of volunteers, and synoptic documents regarding the study were approved by the Scientific Ethics Committee of A.S.L. Napoli 1 CENTRO (Protocol number 222, 12 April 2021) and carried out in accordance with the Helsinki Declaration of 1964 (as revised in 2000). This study is listed on the ISRCTN registry (www.isrctn.com) with ID number 9301859. https://www.isrctn.com/ISRCTN19301859 (accessed on 5 August 2022). 

The study design included two experimental groups (20 subjects for each group). The enrolled subjects were assigned to each of the two groups in a random and unpredictable way by means of a simple randomization (allocation ratio 1:1). At the baseline visit (t0), information on the sociodemographic, clinical, and biochemical characteristics (i.e., body mass index (BMI), total cholesterol (TC), low-density lipoprotein cholesterol (LDL-C), high-density lipoprotein cholesterol (HDL-C), and triglycerides) of the recruited subjects was collected and reported in the case report form (CRF). During the first visit (t0), the recruited subjects initially underwent a fasting blood draw. Then, the recruited subjects subsequently consumed the standard meal, consisting of breadsticks eaten within 15 min with 500 mL of oligomineral water, and BSG extract-based food supplement (group 1) or the standard meal, oligomineral water, and placebo (group 2). Then, blood sampling at timed intervals measured postprandial glycemia and insulinemia (i.e., at 15 (t1), 30 (t2), 60 (t3), 90 (t4), and 120 (t5) min after the intake of breadsticks, water, and the treatment or placebo). This step was followed by a five-day wash-out period (in which the recruited subjects took no treatment), prior to the cross-over of treatments. After the five-day wash-out period, each subject in the two groups underwent blood sampling again (at the times indicated above) for measurement of blood glucose and insulin, before and after ingestion of the standard meal, oligomineral water, and placebo (group 1) or the standard meal, oligomineral water, and the food supplement (group 2), according to the cross-over design. 

Participants were asked to reduce their fiber intake from two weeks before the start of the study until the end, and to not significantly change their eating habits for the entire duration of the study.

#### 2.3.1. Study Population

Forty-subjects aged 18–65 years of either sex were recruited by the general practitioners of Comegen in September 2021. Inclusion criteria included healthy subjects (according to their clinical history), non-smokers, and subjects able to understand and to sign the informed consent. Subjects with type 1 or 2 diabetes, subjects with fasting blood glucose > 110 mg/dL, subjects with blood pressure values > 160/100 mmHg, subjects with metabolic or eating disorders, subjects with disorders that may have interfered with the results of the study (i.e., endocrine, cardiovascular, pulmonary, renal or gastrointestinal diseases), subjects sensitive, intolerant or allergic to the ingredients of the food supplement used in the clinical trial, pregnant or lactating women, blood donors in the three months prior to recruitment, subjects under pharmacological treatment with drugs that could interfere with the study (i.e., alpha-glucosidase inhibitors, insulin-sensitive drugs, sulfonylureas, cholesterol lowering drugs, and any other medications that the physician does not deem compatible with the study), subjects who were taking food supplements that could interfere with the study (i.e., products high in vitamins and minerals (>200% VNR), B vitamins, C vitamin, calcium, zinc, copper, chromium, iodine, iron, magnesium, manganese, phosphorus, essential fatty acid products, botanicals, and any other products that the physician does not deem compatible with the study) were excluded from the study.

#### 2.3.2. Outcomes of the Study

The primary outcome of the present clinical study was to evaluate the contribution of the food supplement based on BSG extract, as part of a standard meal, in promoting the reduction of postprandial blood glucose increase in normoglycemic subjects. The secondary outcome was to evaluate the impact of the food supplement on the postprandial insulinemic response. 

Data collection was performed by means of a CRF divided into two main sections. A first section concerning personal data, subject’s medical history, intake of any concomitant drugs, and the treatment group, filled at the time of enrollment. The second section was filled with the results of the analyses performed on the blood samples taken.

#### 2.3.3. Safety

BSG extract is an approved ingredient for food supplements. Although no adverse events related to the intake of the food supplement were expected, the enrolled subjects were continuously monitored for the occurrence of any kind of adverse effects. The subjects with sensitivity, intolerance, or allergy to gluten or barley were categorically excluded from the study.

### 2.4. Statistical Analysis

Sample size calculation was conducted using three 1-β power values (0.80, 0.95, and 0.99), a significance threshold value of α equal to 0.05, and three effect size values (Cohen’s f = 0.20, 0.25, and 0.30, respectively). Sample size was determined to be 40 participants (20 each group). 

The effect of the treatments on the response variables (blood glucose and insulin) was assessed through a linear mixed model (LMM), where the treatment groups (group A and group B), the measurement times (i.e., immediately after the meal, t0; after 15 min, t1; 30 min, t2; 60 min, t3; 90 min, t4; and 120 min, t5), the order of treatment (first and second) and the age and sex of the subjects were entered into the model as fixed effects. The measurement × treatment and measurement × treatment order interactions were included among the independent variables. The measure × treatment interaction is the key variable for the primary endpoint, as it allows testing whether the trend over the course of the measurements differs for the two treatments. 

With regards to the interaction measurement × treatment order, it is used to check whether trends during the measurement period differ according to the order of administration of the treatments (before or after wash-out). The identity of the subject was evaluated as a random effect, which provided a control for differences among the enrolled subjects. 

Analyses were performed using the lme4 [29] packages in R ver. 4.0.1 [30] (R Foundation for Statistical Computing, Vienna, Austria) and unless otherwise stated, data are reported as means ± standard errors. 

For each subject of groups 1 and 2, the glucose and insulin incremental areas under the curves (iAUCs) were calculated. The iAUCs were evaluated statistically using a *t*-test: two paired samples for means, with each subject being his or her own control. Differences resulting in *p*-values below 0.05 were considered significant.

## 3. Results

### 3.1. Chemical Characterization of BSG Extract

In this study, the extract used was produced from brewer’s spent grain. 

First, the fiber present in the extract was characterized. To determine total soluble and insoluble fiber, the BSG extract was analysed by a gravimetric method involving the elimination of starch and proteins following treatment with α-amylase, amyloglucosidase, and protease, respectively. After this treatment, the BSG extract did not show any precipitate, indicating the absence of insoluble fiber. To determine the soluble fiber, 78% ethanol was added to the sample. The high molecular weight soluble fiber, precipitated and determined by a gravimetric method, resulted to be 7.45 g/100 g BSG extract (Table 2). Since low molecular weight soluble fiber does not precipitate into 78% ethanol, this fiber fraction was not determined with this assay. Therefore, total AXs and β-glucans were determined by hydrolyzing glycosidic bonds with specific enzymes and determining their concentrations on the bases of the concentrations of arabinose and xylose and glucose deriving from their hydrolysis, respectively. The results showed that total AXs and total β-glucans were 7.50 and 1.92 g/100 g of BSG extract, respectively. The same method was also applied to the total fiber dry residue isolated with precipitation under 78% ethanol to determine the content of high molecular weight AXs and β-glucans isolated by the above method. The concentration of AXs was 0.45 g/100 g, but β-glucans were not found to be detectable in the total fiber dry residue. The concentration of low molecular weight AXs soluble in 78% ethanol, and therefore not calculated with the gravimetric method, was calculated by the difference, and resulted to be 7.05 g/100 g of BSG extract. 

Gelling properties of AXs are mainly attributed to WEAX, with AEAX showing less gelling and therefore being less active in the modulation of glucose absorption. WEAX and AEAX were determined to have concentrations of 1.23 g/100 g (representing about 16% of total AXs) and 6.36 g/100 g (representing about 84% of total AXs), respectively. 

Finally, the concentrations of (1) free glucose, (2) glucose derived from rapidly digestible starch and slowly digestible starch (including free glucose), and (3) total glucose deriving from resistant starch (including glucose derived from rapidly digestible starch, slowly digestible starch, and free glucose) were determined in the BSG extract. The concentration of glucose deriving from resistant starch, calculated by the difference between total glucose concentration determined after 4 h of enzymatic treatment, and after 30 min of enzymatic hydrolysis (corresponding to glucose concentration from rapidly digestible, slowly digestible starch, and free glucose), resulted to be 14.64 g/100 g of BSG extract. In addition, total glucose derived from resistant starch was isolated from the total fiber dry residue and resulted to be 0.62 g/100 g of BSG extract. Thus, the concentration of resistant starch soluble in 78% ethanol, and therefore not calculated with the gravimetric method, was calculated by the difference, and resulted to be 14.02 g/100 g of BSG extract. 

In total, the whole BSG extract contained 30.40 g of dietary fiber/100 g of BSG extract, mainly represented by resistant starch (14.64 g/100 g), AXs (7.50 g/100 g), β-glucans (1.92 g/100 g), and other soluble fibers (6.38 g/100 g) isolated with 78% ethanol precipitation and calculated by the difference between total soluble fiber dry residue (7.45 g/100 g) and high molecular weight AXs (0.45 g/100 g) and resistant starch (0.62 g/100 g) weights.

**Figure 1:**
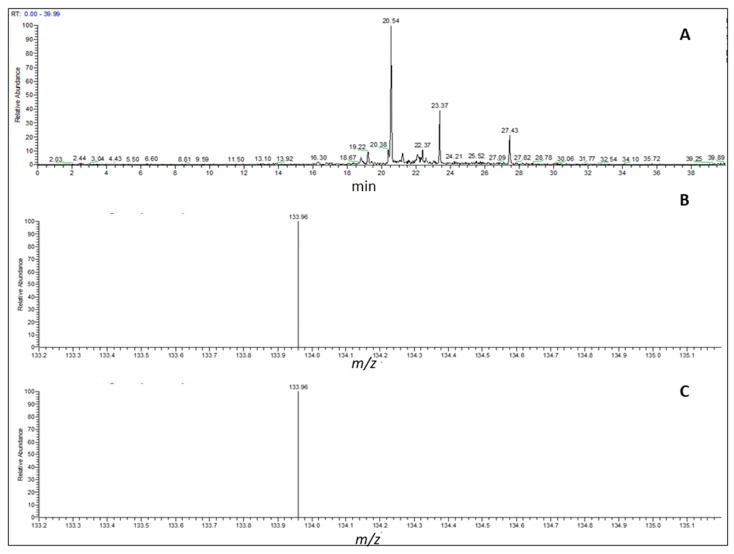
The chromatogram of an ion product with m/z 193 obtained from the HUPLC-MS/MS analysis of digested BSG extract (**A**). Chromatogram recorded at 320 nm; (**B**). Mass spectrum of parent ion with *m/z* 193; (**C**). Mass spectrum of fragmentation of parent ion with *m/z* 193. LOQ and LOD values determined for ferulic acid were 0.062 and 0.016 μg/mL, respectively.

TPC before and after oral-gastric-duodenal digestion were found to be 0.499 ± 0.01 and 1.16 ± 0.03 g GAE/100 g of BSG extract, respectively, suggesting that during digestion polyphenols are released by the food matrix and become bioaccessible. As spectrophotometric methods generally have issues with overestimating the phenolic contents, since Folin–Ciocalteu reagent interacts with non-polyphenolic molecules (i.e., reducing sugars), the ferulic acid content, which is the polyphenol most represented in BSG, was evaluated by means of a validated UHPLC-MS/MS method after oral-gastric-duodenal digestion [31]. Its identification was based on the mass spectrum and fragmentation pattern of the parent ion with m/z 193 (Figure 1). The content of ferulic acid was 64.8 ± 0.06 mg/100 g of digested BSG extract, corresponding to 91.3 ± 0.07 mg/100 g of BSG extract.

### 3.2. Clinical Trial

**Figure 2:**
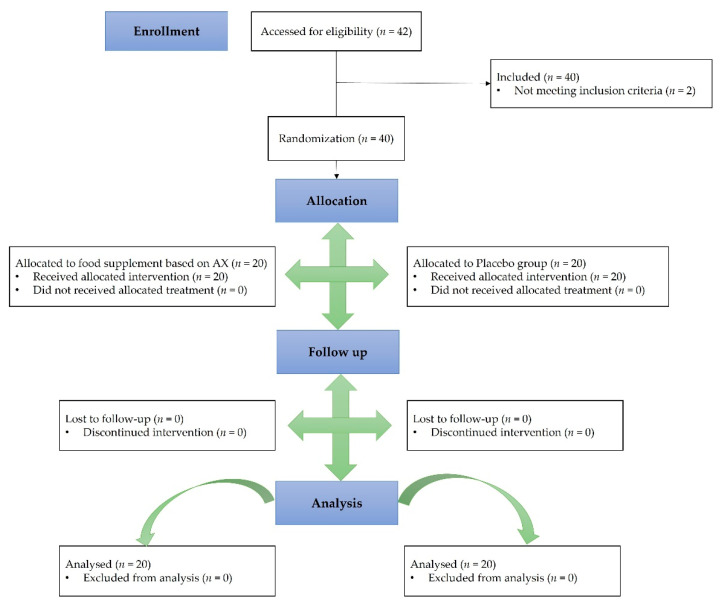
CONSORT Flow diagram.

The study flow chart, produced in accordance with the CONSORT PRO reporting guidelines [32], is shown in Figure 2. Initially, 42 subjects were screened for the clinical study; however, two of these subjects did not meet the inclusion criteria and therefore were excluded. The total number of subjects enrolled was 40, and they were randomly assigned to either group 1, receiving the BSG extract-based food supplement (treatment A) first and then the placebo (treatment B), or to group 2, which first received the placebo and then the BSG extract-based food supplement. As the aim of the clinical trial is to unravel the efficacy of BSG extract in reducing post-prandial glycemia and improving insulinemic response, the BSG extract-based food supplement was compared with the placebo consisting of indigestible carbohydrates. Group 1 consisted of 13 women (65%) and 7 men (35%), and group 2 consisted of 12 women (60%) and 8 men (40%). 

The participants in the two groups had similar sociodemographic characteristics and clinical data, with no significant differences with the exception of HDL-C (*p* = 0.01). The baseline characteristics of the subjects for each group are summarized in Table 3.

The primary objective of the study was to evaluate the contribution of BSG extract-based food supplement in promoting the reduction of postprandial blood glucose in normoglycemic subjects with slightly impaired glucose tolerance, shown by HOMA-IR Index and Triglycerides and Glucose index (TyG) values higher than 2.5 and 4.5, respectively, for most of the recruited subjects [33] (Table 3). In fact, the upper limits of the ranges for triglycerides, HDL-C, and LDL-C greater than 150 mg/dL), lower than 40 mg/dL, and higher than 159 mg/dL, respectively, lead to thinking that part of the subjects recruited has an altered lipid profile compatible with mild insulin resistance [34] (Table 3). Nevertheless, at the baseline (t0) all 40 subjects were normoglycemic (Table 4).

Glycemia and insulin levels for the two study groups, at different time points, both in male and in female subjects, are reported in Table 4. As expected, the mean postprandial glycemia values recorded after 15 and 30 min tended to grow to a peak at 60 min, regardless of sex and treatment. After peaking at 60 min, the mean postprandial glycemia values recorded after 90 and 120 min tended to decrease more in the subjects who had taken the food supplement than those subjects who had taken the placebo, regardless of sex. In fact, while from 0 to 60 min the post-prandial glycemia values of the subjects (both male and female) who had taken the food supplement and the placebo were overlapping, at 90 and 120 min, the mean postprandial glycemia values of the subjects who had taken the food supplement were lower than the corresponding values of the subjects who had taken the placebo. As far as postprandial blood insulin is concerned, the mean post-prandial insulinemia values recorded after 15 and 30 min tended to grow to a peak of 60 min in the placebo group more than in the food supplement group, regardless of sex. After the peak recorded at 60 min, also in this case the average post-prandial insulinemia values recorded after 90 and 120 min tended to decrease more in the subjects who had taken the food supplement than in those who had taken the placebo, regardless of gender.

**Figure 3:**
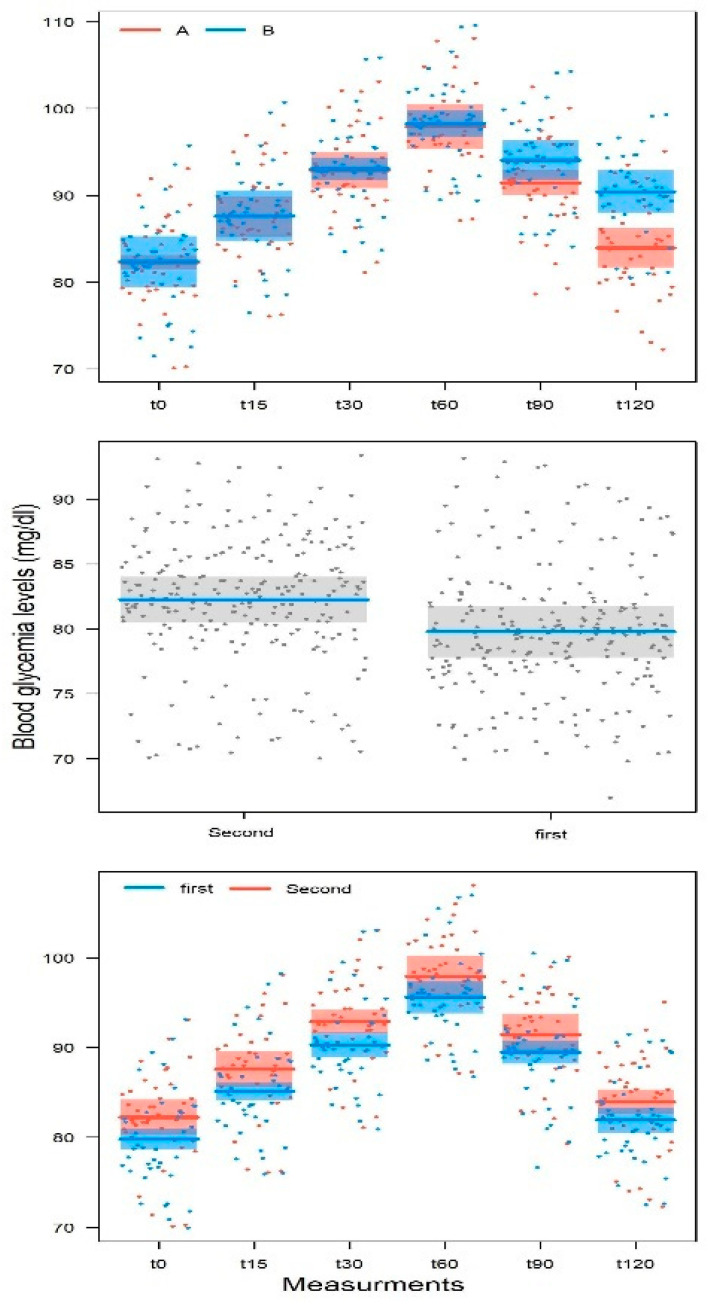
Variation in postprandial glycaemia for the two experimental treatments. **Above**: variation in blood glucose for the two experimental treatments (A: BSG extract-based food supplement and B: placebo); **middle**: blood glucose values before and after wash out; **below**: change in blood glucose before and after wash out regardless of experimental treatment.

The LMM model for blood glucose (Table 5) identified a statistically significant effect for the measurement (*p* < 0.001), for the treatment (*p* < 0.001), and also for the measurement × treatment interaction (*p* < 0.001). Significant effects also emerged for sex (*p* = 0.014) and age (*p* = 0.032). Furthermore, the effect of the treatment order was also significant (*p* < 0.001); the measurement × order of treatment was not (*p* = 0.99). These results indicate that there was a difference between treatments A and B when it came to the postprandial glycemic curve of patients (Figure 3—top figure). Blood glucose did not differ between treatments, from the initial measurement to the peak at 60 min (t0: dB-A = 0.29 ± 1.21, t424 = 0.240, *p* = 0.81; t15: dB-A = 0.22 ± 1.21, t424 = 0.185, *p* = 0.85; t30: dB-A = 0.34 ± 1.21, t424 = 0.283, *p* = 0.77; t60: dB-A = 0.46 ± 1.21, t424 = 0.383, *p* = 0.70). Actually, the blood glucose values of the subjects treated with the food supplement (treatment A) or placebo (treatment B), recorded at t0, t1, t2, and t3, did not change. Differently, the blood glucose values recorded at t4 (90 min) and t5 (120 min) during treatment A were significantly lower than the corresponding values during treatment B in the subsequent phase of descent from the peak, respectively, after 90 min (dB-A = 2.67 ± 1.21, t424 = 2.199, *p* = 0.028) and 120 min (dB-A = 6.55 ± 1.21, t424 = 5.397, *p* < 0.001). 

The trend of the glycemic curve, however, was not different before and after the washout period, as evidenced by the fact that the interaction between measurement and treatment order was not significant (Table 5) (Figure 3—middle and below figure). 

The effect of sex indicates that the men selected in the sample had blood glucose values higher than those of women (4.12 ± 1.60, t36 = 2.575, *p* = 0.014). This difference, however, has no clinical relevance, as the blood glucose values of the subjects at t0 were in line with the inclusion criteria. 

Finally, as far as the age of recruited subjects is concerned, blood glucose tended to increase with age (0.26 ± 0.11, t36 = 2.228, *p* = 0.032) regardless of gender. Moreover, in this case, this difference has no clinical relevance as the blood glucose values of the subjects at t0 were in line with the inclusion criteria. 

The LMM model for insulin (Table 5) provided similar results to that for glycemia. The effects of measurement (*p* < 0.001), treatment (*p* < 0.001), and measurement × treatment interaction (*p* < 0.001) were statistically significant. There was no significant effect for the sex (*p* = 0.25) and age (*p* = 0.12). 

**Figure 4:**
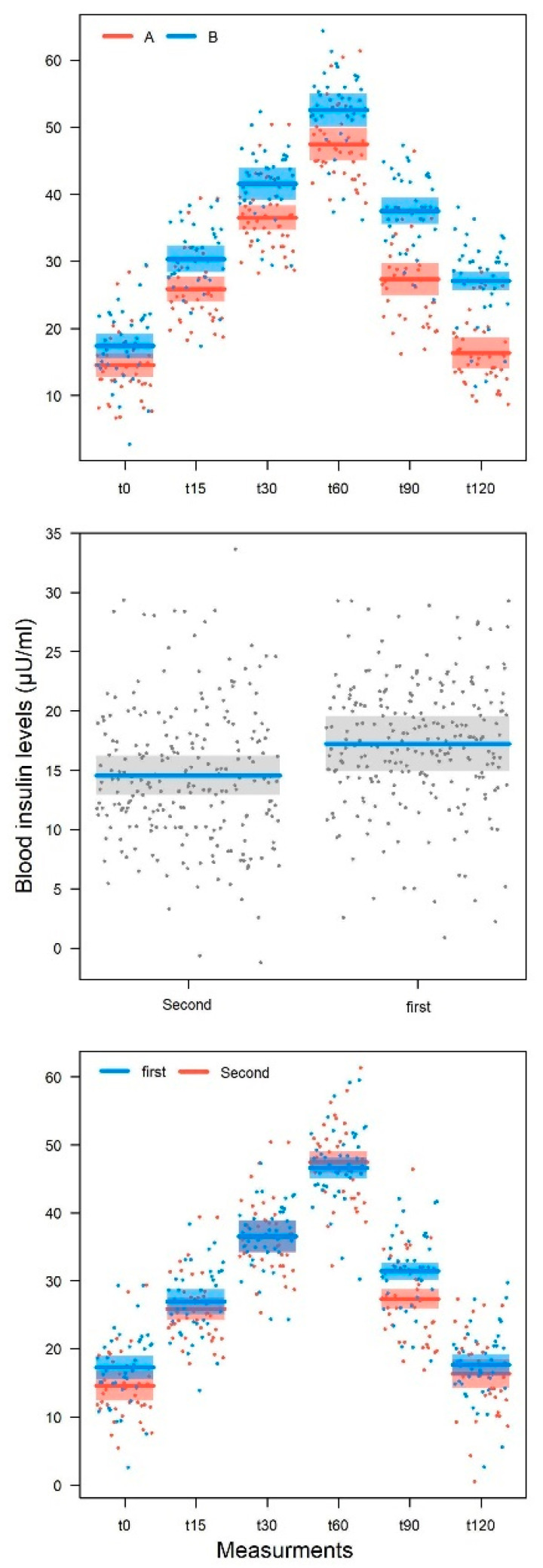
Variation in postprandial insulin values for the two experimental treatments. **Above**: variation of the insulin concentration for the two experimental treatments (A: BSG extract-based food supplement, and B: placebo); **middle**: insulin concentration values before and after washout; **bottom**: change in insulin concentration before and after washout regardless of experimental treatment.

The effect of the treatment order was also statistically significant in this case (*p* = 0.009), but the interaction between measurement and treatment order (*p* = 0.10) was not such. These results indicate that there is a difference in the postprandial insulin curve in patients undergoing treatment A versus those when undergoing treatment B (Figure 4—top figure). 

The insulin values differed between treatments, since the initial measurement (dB-A = 2.83 ± 1.31, t424 = 2.169, *p* = 0.031) increased at 15 min (dB-A = 4.51 ± 1.31, t424 = 7.077, *p* < 0.001) and gradually at 30 min (dB-A = 5.02 ± 1.31, t424 = 7.590, *p* < 0.001), reached a peak at 60 min (dB-A = 5.10 ± 1.31, t424 = 7.650, *p* < 0.001) and 90 min (dB-A = 10.16 ± 1.31, t424 = 12.733, *p* < 0.001), with a maximum measurement at 120 min (dB-A = 10.79 ± 1.31, t424 = 13.336, *p* < 0.001). 

This order indicates that the insulin value after the washout is significantly lower than that observed before the washout (−1.39 ± 0.53, t423 = 2.612, *p* = 0.009), regardless of the experimental treatment. As for glycaemia, the trend of the curve was not different between before and after the washout period for insulinemia, as evidenced by the fact that the measurement × treatment order was not significant (Table 5).

The incremental areas under curve (iAUCs) of postprandial glycemia of the subjects treated with BSG extract-based food supplement and the corresponding iAUCs of the subjects taking placebo did not show any statistical difference, while the mean incremental area under curve (iAUC) of insulinemia of the subjects treated with BSG extract-based food supplement (iAUC = 1928 ± 237) were 19.7% significantly lower (f-ratio value = 6.30397, *p*-value = 0.013436) than the corresponding mean iAUC of the subjects taking the placebo (iAUC = 2457 ± 400). 

As far as safety is concerned, none of the subjects reported any adverse event after receiving the food supplement.

## 4. Discussion

In this study, a new extract from brewer’s spent grains was used as a bioactive ingredient for food supplements and tested for its effects on postprandial glycemia and insulinemic response in a monocentric, randomized, cross-over, double-blind, placebo-controlled clinical trial. 

Although the literature data report that the main fiber families present in BSG are insoluble fibers (i.e., cellulose, lignin, and hemicellulose) [19], the BSG extract used in this study does not contain insoluble fiber. This composition is probably due to the patented enzymatic extraction method that allows enrichment of the extract with soluble fibers [35]. In agreement with the literature data [19], this BSG extract consists of resistant starch (14.6 g/100 g), followed by AXs (7.5 g/100 g) and β-glucans (1.9 g/100 g). Among AXs, 5% and 95% of the total AX content are represented by high molecular weight and low molecular weight AXs, respectively. Moreover, the water extractable AXs represent about 16% and alkali extractable AXs represent about 84% of total AX content. The major gelling properties, which, in turn, are responsible for the lower and slower absorption of glucose from the diet, are mainly ascribed to water-soluble, high molecular weight AXs. Based on the obtained results regarding AXs, we expect the BSG extract effect on glucose absorption to be modest.

On the basis of the results of the analyses of the different kind of fiber of BSG extract, which was found to contain about 31 g of soluble fiber per 100 g of BSG extract, one stick pack of the BSG extract-based food supplement, at 5.0 g/stick pack of BSG extract, contains about 1.5 g of soluble fibers and 0.75 g of inulin (about 7% of the recommend consumption of 25 g for adult women and 38 g for adult men, based on epidemiologic studies showing protection against cardiovascular disease) [36]. 

Most of the phenolic compounds present in barley grains are found in the husk, and thus BSG represents a rich source of polyphenols. The TPC obtained from the analysis of BSG extract before the in vitro simulated digestion process resulted to be 0.49 g GAE/100 g. This value is comparable to those reported by Birsan et al. that found that TPC of the crude extract ranged from 0.28 to 0.38 g GAE/100 g, and by Meneses et al. that reported that TPC of the BSG extracted with water resulted to be 0.39 g GAE/100 g [37,38]. After the in vitro simulated digestion process, TPC was found to be 1.16 g GAE/100 g. Although this result is an overestimation of polyphenol content, it is however indicative of the presence of bioaccessible polyphenols that are not degraded by digestion and that can perform their beneficial effects at the intestinal level. 

Ferulic acid is the main free and bound polyphenol in barley, malt, and BSG [39]. As ferulic acid has been shown to exert anti-diabetic effects in many in vitro and in vivo studies through different mechanisms of action (i.e., reduction of oxidative stress in pancreatic islets, which, in turn, causes their necrosis leading to reduced secretion of insulin, improvement in the activities of antioxidant enzymes (i.e., superoxide dismutase and catalase) in the pancreatic tissue, and increase of glucose uptake in insulin resistant cells) [40], to understand how much ferulic acid remains available to exert its beneficial biological activities after digestion, the residual free ferulic acid content after in vitro simulated oral-gastric-duodenal digestion was determined. Ferulic acid concentration was found to be about 90 mg/100 g BSG extract. The ferulic acid content of BSG depends on the varieties of barley used, the malting and brewing processes, and the extraction method used to treat the sample before analysis. Mussatto et al. showed that by applying optimized alkaline hydrolysis conditions for the extraction of ferulic acid from BSG, its concentration resulted to be 9.65 mg/ per gram of solubilized lignin, corresponding to 286 mg/100 g of BSG [41]. The result obtained from the analysis performed in the present investigation showed a lower concentration of released ferulic acid in comparison with the result obtained by Mussatto et al. This result can be due to variations in the treatment of the samples that lead to different yields in the release of ferulic acid. Nevertheless, we decided to apply a simulated in vitro digestion process as it is more in line with the human digestive process and can give a more accurate view of the bioaccessibility and bioavailability of ferulic acid. 

Regarding the results of the clinical trial on postprandial glycemia and insulinemia, a large body of evidence suggests that postprandial hyperglycemia is a risk factor for the onset of diabetes and cardiovascular disease [42,43].

The results of this clinical trial showed that blood glucose values measured after BSG extract-based food supplement intake were significantly lower than the corresponding values for the placebo group only in the descent phase of the glucose peak, respectively, after 90 min and 120 min, while at the baseline (t0) and in the first 60 min (t1–t3), the two glycemic curves overlapped substantially. We justify the absence of an effect of the intake of the food supplement taken with the standard meal on post-prandial blood glucose recorded in the first 60 min, with the low content and poor gelling properties of soluble fibers, especially AXs, present in BSG extract, that do not induce a significant reduction in glucose absorption. In addition, the glycemic curves of the subjects who took a placebo have trends typical of those curves recorded after the consumption of a starchy food. In fact, as reported by Brand-Miller et al. [44], starchy foods provide more glucose than sugary foods (i.e., soft drinks and fruit juices), and therefore were generally more likely to produce a curve that remained above baseline at 120 min. The intake of BSG extract-based food supplement, while not inhibiting the absorption of glucose, in the first 60 min, probably slightly reduces and slows down the absorption of glucose in accordance with the fiber content of the food supplement. The explanations of the modest recorded effect on postprandial glycemia produced by BSG extract based-food supplement may be due at least in part to the presence in the BSG extract of simple sugars, which increase glycemia, and to the fact that the effect of the food supplement is compared with the placebo that contains 15% inulin, which is a dietary fiber known to have hypoglycemic effects [45,46].

However, since the purpose of the clinical study was to test the effect of BSG extract, to exclude the effect of inulin used as carrier agent, the same amount of inulin present in the food supplement was added to the placebo. Although modest, the intake of the BSG extract based-food supplement produced a beneficial effect on glycemia as postprandial glycemic curve returned to baseline earlier. This improved clinical outcome was corroborated by significant reductions in postprandial insulinemic response. In particular, the blood insulin values of the subjects that took the food supplement were significantly lower from the first 15 min, with growing differences that reached maximum difference at 120 min from the first blood sample (t0). The mean insulin iAUC of the subjects who took the BSG extract-based food supplement was 19.7% significantly lower than the iAUC of the subjects taking placebo (*p* < 0.05). On the whole, these results mean that acute intake of BSG extract-based food supplement induces an improvement in postprandial insulinemic response. Moreover, it is worth noting that although the mean values of glycemia, BMI, and blood lipids of the recruited subjects are normal, however, on the basis of fasting glycemia, insulinemia, and triglyceridemia, most subjects showed a mild insulin resistance, as evidenced by HOMA Index and TyG values higher than 2.5 and 4.5, respectively. Thus, BSG extract-based food supplement, while not leading to a lowering of the postprandial glycemic curve, improved insulinemic response in subjects with mild insulin resistance. 

Moreover, in accordance with the literature data, we found blood glucose values were significantly higher in men than in women irrespective of both the six measures the two experimental treatments, although these differences have no clinical relevance [47,48,49,50], and blood glucose tended to increase with age, independently from gender. It is well known that aging is associated with increased fasting blood glycemia due to a reduction in glucose-induced insulin release, and increased inflammation markers, which, in turn, increase insulin resistance in muscle and adipose tissue [51]. 

This work has limitations and strengths. The main limitations are represented by the fact that, due to the acute nature of this clinical trial, the effect of continuous ingestion of BSG extract and the influence of its fermentation products on glucose metabolism could not be determined, making it impossible to learn about any longer-term effects of this supplementation. Secondly, its effect on diabetic and pre-diabetic patients is unknown, as the subjects of this clinical trial were limited to normo-glycemic subjects showing just mild insulin resistance. Finally, the presence in the food supplement of simple sugars, which together with the standard meal contribute to the rise of postprandial blood glucose, and the absence of an equal number of simple sugars in the placebo probably cause an underestimation of the actual properties of BSG extract-based food supplement to reduce the increase in post-prandial glycemia. 

On the other hand, the major strength of this study is that the fiber and polyphenol composition of the BSG extract-based food supplement is known, and therefore the recorded effects on postprandial blood glucose and insulinemic response can be linked to the simultaneous presence of resistant starch, AXs, and β-glucans.

## 5. Conclusions

In conclusion, there is considerable importance in reducing postprandial glucose and insulin increases responsible for oxidative stress and β cells damage, which in turn are considered risk factors for serious chronic diseases. There is also a growing interest in the development of new economically and environmentally sustainable food products designed to improve glucose metabolism. The BSG extract-based food supplement tested, containing soluble dietary fiber and bioaccessible ferulic acid, was found to lead to the restoration at 120 min of blood glucose and insulin values to the values recorded at the baseline, reducing postprandial glucose and insulin increases in normo-glycemic subjects showing just a mild insulin resistance. 

More studies are required to determine whether these acute benefits result in long-term improvements in glycemic control and whether supplementation with this food supplement as part of the daily diet is a useful approach for the management of subjects with slight insulin resistance. If these promising results are confirmed in long-term studies, this food supplement might be recommended to elderly people at a high risk of developing metabolic syndrome and diabetes (i.e., with pre-diabetes, overweight, 45 years or older, have a parent, brother, or sister with type 2 diabetes, are physically active less than 3 times a week) and women of childbearing age with risk factors towards developing gestational diabetes during pregnancy.

## Figures and Tables

**Table 1:** Breadsticks nutritional values.

Average Nutritional Values	g/100 g of Product
Energy	1601 kJ–378 kcal
Fats	2.5 g
of which saturated fatty acids	0.5 g
Carbohydrates	74.5 g
of which sugars	3.4 g
Dietary fibers	4.8 g
Protein	12.7 g
Salts	1.3 g

**Table 2:** Concentrations of free glucose, arabinose, and xylose, total AXs, WEAX and AEAX, total β-glucans, glucose from rapidly and slowly digestible starch, and glucose from resistant starch, in BSG extract, and AXs, β-glucans, and resistant starch determined in the total fiber dry residue.

Compound	Concentration (g/100 g) ^1^
Free glucose occurring in BSG extract	5.53 ± 0.01
Free arabinose occurring in BSG extract	0.71 ± 0.01
Free xylose occurring in BSG extract	0.95 ± 0.01
Total AXs occurring in BSG extract	7.50 ± 0.05
WEAX—water extractable arabinoxylans	1.23 ± 0.02
AEAX—alkali extractable arabinoxylans	6.36 ± 0.03
AXs insoluble in ethanol (78%) occurring in total fiber dry residue	0.45 ± 0.01
AXs soluble in ethanol (78%) ^2^	7.05 ± 0.01
Total β-glucans occurring in BSG extract	1.92 ± 0.05
β-glucans insoluble in ethanol (78%) occurring in total fiber dry residue	N.D.^3^
Glucose after 30 min of enzymatic hydrolysis occurring in BSG extract ^4^	30.36 ± 0.01
Glucose after 4 h of enzymatic hydrolysis occurring in BSG extract ^5^	45.00 ± 0.06
Glucose after 4 h of enzymatic hydrolysis occurring in total fiber dry residue	0.62 ± 0.01
Total dietary fiber	7.45 ± 0.03

^1^ Data expressed as means ± SD (*n* = 3). ^2^ calculated by the difference between total AXs present in the BSG extract and AXs present in the total fiber dry residue. ^3^ N.D. not detectable. ^4^ corresponding to glucose from rapidly and slowly digestible starch, including free glucose. ^5^ corresponding to glucose from resistant starch, including glucose from rapidly and slowly digestible starch and free glucose.

**Table 3:** Characteristics of the study population: demographic and clinical data at baseline (t0).

Features	Group 1 (*n* = 20)	Group 2 (*n* = 20)
Age (years)	53 ± 5	57 ± 7
Gender:		
Male	7	8
Female	13	12
Ethnicity: European	20	20
BMI (kg/m^2^)	21.82 ± 2.05 − (18.6–24.7)	21.93 ± 2.32 − (18.5–24.8)
TC (mg/dL)	217.25 ± 17.89 − (180–245)	220.7 ± 17.71 − (180–246)
HDL-C (mg/dL)	48.55 ± 12.14 − (30–65)	54.9 ± 9.18 − (33–68)
LDL-C (mg/dL)	112.85 ± 17.78 − (83–153)	120.25 ± 23.42 − (82–160)
Triglyceride (mg/dL)	131.55 ± 26.49 − (82–167)	125.6 ± 22.6 − (85–170)
Homa index	3.39 ± 1.32 − (1.21–5.25)	4.58 ± 1.45 − (1.54–6.81)
TyG index	8.51 ± 0.24 (7.99–8.86)	8.61 ± 0.21 (8.19–8.99)

**Table 4:** Variation in values (mean ± standard deviation, minimum and maximum) of blood glucose and insulin in men and women for the two experimental treatments (A: BSG extract-based food supplement, and B: placebo).

Variable Treatment		t0	t1	t2	t3	t4	t5
Blood glycemia (mg/dL)							
Female	A	81.6 ± 7.7	86.9 ± 7.6	92.2 ± 7.8	97.5 ± 7.6	91.4 ± 7.3	83.3 ± 8.3
		(70–95)	(75–100)	(80–105)	(85–110)	(80–106)	(72–101)
	B	81.5 ± 6.5	86.7 ± 6.4	91.8 ± 6.5	97.3 ± 6.6	93.2 ± 6.7	89.3 ± 6.5
		(70–94)	(76–99)	(81–104)	(87–109)	(82–105)	(79–101)
Male	A	86 ± 8.1	91.3 ± 7.9	96.4 ± 7.8	101.4 ± 7.4	94.8 ± 8.1	88.3 ± 8
		(72–95)	(77–100)	(83–106)	(88–110)	(82–105)	(74–96)
	B	84.3 ± 7.6	89.7 ± 7.4	95.3 ± 7.1	100.5 ± 6.7	96.5 ± 6.6	93.2 ± 6
		(74–94)	(80–100)	(86–105)	(91–109)	(85–104)	(83–100)
Blood insulin (µU/mL)							
Female	A	17.3 ± 7.2	27.7 ± 7.2	37.8 ± 7	48.3 ± 7	30.5 ± 9.6	18.5 ± 7.1
		(6–30)	(17–41)	(27–52)	(38–62)	(18–48)	(8–34)
	B	17.9 ± 8.1	30.2 ± 7.5	40.6 ± 7.7	51.3 ± 8.5	38.1 ± 7.7	26.8 ± 8.8
		(6–30)	(20–41)	(28–53)	(34–64)	(25–51)	(9–39)
Male	A	16.3 ± 6.4	26.9 ± 6.2	36.9 ± 6.5	47.4 ± 6.1	30.3 ± 6.8	17 ± 5.4
		(7–28)	(18–39)	(28–49)	(39–58)	(20–42)	(9–27)
	B	21.6 ± 6	33.7 ± 6.7	44.8 ± 6.9	55.2 ± 7.9	43.2 ± 5.7	30.9 ± 5.8
		(10–30)	(21–45)	(32–58)	(43–72)	(33–51)	(20–40)

**Table 5:** LMM model results for glucose and insulinemic response curves.

Template	F	Df	P
**Glycemia**			
Measurement	89.11	5, 423	**<0.001**
Treatment	11.01	1, 427	**<0.001**
Sex	6.63	1, 37	**0.014**
Age	4.96	1, 37	**0.032**
Processing order	22.83	1, 423	**<0.001**
Measurement × Treatment	4.69	5, 423	**<0.001**
Measurement × Order of treatment	0.04	5, 423	0.99
**Insulin**			
Measurement	324.06	5, 423	**<0.001**
Treatment	138.95	1, 428	**<0.001**
Sex	1.36	1, 37	0.25
Age	2.58	1, 37	0.12
Processing order	6.87	1, 423	**0.0097**
Measurement × Treatment	6.29	5, 423	**<0.001**
Measurement × Order of treatment	1.86	5, 423	0.10

## Data Availability

Not applicable.
